# Lysosomal-stress-driven cargo-fate switching between autophagic degradation and small extracellular vesicle release in triple-negative breast cancer: a hypothesis and theory article

**DOI:** 10.3389/fonc.2026.1923547

**Published:** 2026-09-21

**Authors:** Yuanpeng Liu, Guocui Qi, Anjun Wang, Fengxiao Hu

**Affiliations:** 1 School of Medicine, Qilu Institute of Technology, Jinan, Shandong, China; 2 School of Medicine, Shandong Xiehe University, Jinan, Shandong, China

**Keywords:** autophagy, lysosomal stress, RASAL2, RIPK1, secretory autophagy, small extracellular vesicles, triple-negative breast cancer

## Abstract

Triple-negative breast cancer (TNBC) cells face metabolic, proteotoxic and treatment-associated stress, but the mechanisms that determine how stressed cells dispose of intracellular cargo remain incompletely defined. We hypothesize that lysosomal stress shifts part of the autophagy-associated cargo burden from intracellular degradation towards extracellular release in small extracellular vesicle (sEV)-enriched fractions. The rationale integrates general evidence linking autophagy with EV biology with two model-specific observations: RIP1 involvement in Manzamine A-induced secretory autophagy in breast cancer cells and RASAL2-dependent modulation of Rab27a activity and autophagy-associated sEV release in MDA-MB-231 cells. These observations motivate the hypothesis but do not establish its generality across TNBC backgrounds or forms of lysosomal stress. We therefore regard RIPK1 as a candidate regulator whose role may depend on the stress context, whereas the RASAL2-Rab27a module is functionally supported but mechanistically unresolved. The model predicts that perturbing these components will alter time-resolved paired intracellular and extracellular measurements. A coordinated sequence of lysosomal and autophagic changes preceding vesicle-associated export, together with exclusion of cell-death-associated particle release, would support the model; stressor-restricted, arm-specific or injury-associated outcomes would require its revision.

## Introduction

Triple-negative breast cancer (TNBC) accounts for approximately 10% to 20% of breast cancers and remains clinically challenging because of early recurrence, metastatic dissemination and therapeutic resistance (1). Immune checkpoint inhibitors, poly(ADP-ribose) polymerase inhibitors and antibody-drug conjugates have improved outcomes in selected settings, but durable disease control remains uneven (2, 3). This gap suggests that TNBC cells continue to rely on adaptive stress-response programs that help them tolerate metabolic pressure, therapy-induced injury and a fluctuating tumor microenvironment (2–5).

A central stress-adaptation problem is how tumor cells handle intracellular cargo when lysosomal degradation becomes limiting. Autophagy, multivesicular body dynamics, lysosomal fusion and extracellular vesicle (EV) secretion share endolysosomal checkpoints (6–14). When lysosomal degradation is efficient, autophagy-associated cargo is mainly cleared inside the cell. When lysosomal competence is impaired, part of this cargo may accumulate or be redirected towards extracellular release, including release in sEV-enriched fractions (8–10, 15–22).

TNBC-derived EVs can transfer proteins, nucleic acids and membrane-associated cargo to tumor, stromal and immune cells, thereby influencing immune regulation, invasion, metastasis and treatment response. Circulating EV-associated cargo is also being explored as a potential liquid-biopsy readout (23–28). These biological and clinical roles make it important to distinguish regulated vesicle release from nonspecific extracellular particle accumulation during cell injury. Interpretation nevertheless requires care because isolation methods recover heterogeneous vesicle populations and endosomal origin is often not directly demonstrated (11, 13, 14, 29). In accordance with MISEV guidance, we therefore use EV and sEV as operational terms and treat sEV-enriched cargo as a readout rather than proof of a single biogenetic route (29).

Against this background, the unresolved question is whether lysosomal dysfunction, RIPK1 signaling and RASAL2/Rab27a-dependent vesicle release can be linked by a common, testable cargo-allocation model in TNBC.

## Central hypothesis

The central hypothesis is that lysosomal stress creates a cargo-fate state in TNBC in which autophagy-associated material is incompletely degraded and partly redirected towards extracellular disposal in sEV-enriched fractions. RIPK1 is proposed as a candidate, context-dependent convergence point rather than a general sensor of lysosomal stress. The RASAL2-Rab27a module is functionally supported in a defined TNBC model, although its biochemical mechanism remains unresolved (30–34). This formulation is also consistent with evidence that LC3-associated machinery can influence EV cargo loading and that MVB proteins can be routed towards lysosomal degradation rather than secretion (10, 19). The proposed cargo-fate switching model is illustrated in **Figure 1**.

**Figure 1:**
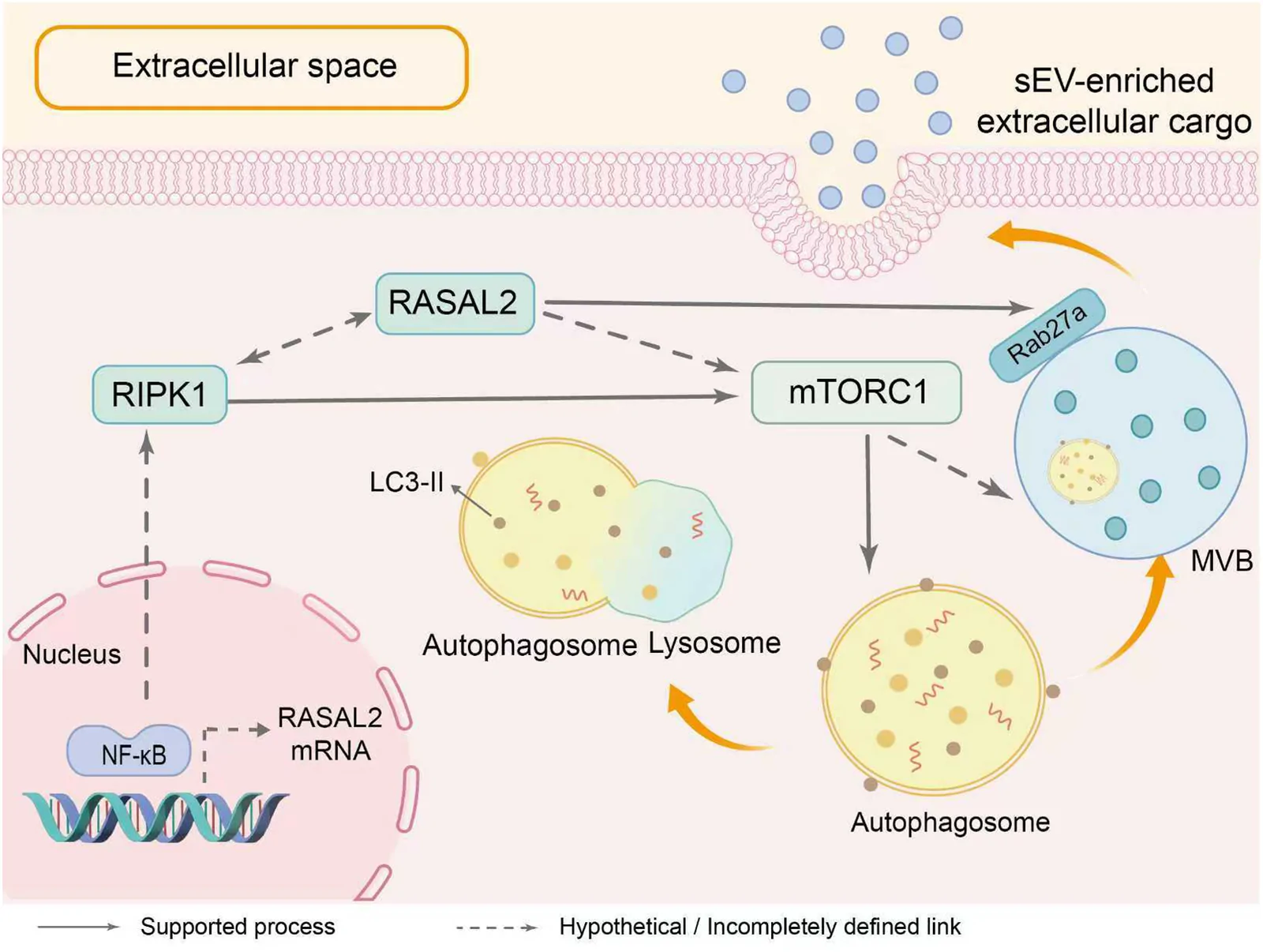
A cargo-fate switching model linking lysosomal stress, autophagic processing and sEV-enriched extracellular disposal in TNBC. The schematic depicts a stress-responsive cargo-fate switching model between intracellular degradation and extracellular cargo release. When lysosomal competence is preserved, autophagyassociated cargo is preferentially delivered for degradation. When lysosomal capacity becomes limiting, part of the cargo burden may be redirected towards extracellular release in sEV-enriched fractions. Solid arrows denote processes supported within the cited experimental model and do not imply general applicability. The solid RASAL2-Rab27a connection denotes functional regulation and biochemical association observed in the cited TNBC model; it does not imply demonstrated direct binding or direct GAP activity. Dashed arrows indicate hypothetical or incompletely defined links. EV, extracellular vesicle; sEV, small extracellular vesicle; TNBC, triple-negative breast cancer.

The hypothesis allows three non-exclusive mechanisms. First, RIPK1-dependent stress signaling may influence RASAL2 expression through a transcriptional program. RIPK1 can engage NF-kappaB-associated transcriptional signaling (35), but a direct RIPK1-to-RASAL2 transcriptional link has not been demonstrated. Second, lysosomal stress may alter RASAL2 phosphorylation, localization, stability or binding partners (32, 33). Third, RIPK1 and RASAL2 may influence lysosomal and vesicular homeostasis through distinct proximal processes that converge on shared outputs, including through TFEB- and mTORC1-linked lysosomal control (21, 22, 36, 37).

These possibilities generate different experimental dependencies. A transcriptional mechanism predicts that changes in RASAL2 mRNA precede changes in protein abundance and downstream cargo handling. A post-translational mechanism predicts altered RASAL2 phosphorylation, localization, stability or binding partners without a corresponding change in transcript abundance. Functional convergence predicts that RIPK1 perturbation need not consistently alter RASAL2 expression, but that factorial perturbation of the two proteins produces shared or interacting effects on lysosomal competence, autophagic flux and vesicle-associated export.

Accordingly, EV abundance alone is insufficient, and transcriptional correlation between RIPK1, RASAL2 and vesicle-associated genes is not mechanistic evidence. Testing the model requires flux-based autophagy assays, measurements of lysosomal competence and MISEV-aligned EV characterization rather than static marker measurements alone (11, 13, 14, 20, 29).

## Rationale for the hypothesis

The first rationale is the established intersection between autophagy and EV release. Autophagosomes, multivesicular bodies and lysosomes use overlapping membrane-trafficking machinery, and lysosomal stress can redirect material towards secretory autophagy-related outputs (6–19). These routes are not interchangeable, but they may become functionally coupled when degradative capacity is constrained.

The second rationale comes from breast cancer and TNBC studies. EVs derived from breast cancer cells can carry biologically active proteins, nucleic acids and membrane-associated cargo that influence neighboring cells and the tumor microenvironment (10, 13, 14, 23, 28). A lysosome-low/export-high state could therefore relieve intracellular stress while altering extracellular signaling.

The third rationale is molecule-specific. In Manzamine A-treated MDA-MB-231 and MCF-7 breast cancer cells, reduced RIP1 expression was associated with AKT/mTOR-related autophagy changes, impaired lysosomal acidification and release of autophagy-associated proteins in sEV fractions; RIP1 overexpression attenuated the autophagy-associated and sEV phenotypes (30). This observation supports RIP1 involvement in that drug-defined model but does not establish RIPK1 as a general controller of lysosomal stress.

In MDA-MB-231 cells, RASAL2 loss altered autophagic flux, lysosomal acidity and sEV release (31). Previous work also demonstrated endogenous and exogenous co-immunoprecipitation of RASAL2 and Rab27a, RASAL2-dependent differences in Rab27a-GTP abundance, and functional effects of combined RASAL2 and Rab27a perturbation on autophagy-associated sEV release (31). These findings support biochemical association and functional coordination. They do not, however, establish direct binary binding or demonstrate that Rab27a is a direct GAP substrate of RASAL2. Separate studies link RASAL2 phosphorylation to breast cancer progression and autophagy-related regulation (32, 33), but do not resolve this relationship.

The conceptual advance of the present article does not lie in re-reporting either observation. It lies in asking whether these findings represent components of a shared, stress-responsive allocation of autophagic cargo between lysosomal degradation and extracellular release, with RIPK1 proposed as a candidate convergence point. A linear RIPK1-RASAL2-Rab27a pathway has not been demonstrated.

## Falsifiable predictions

The model is useful only if it can fail. Its strongest prediction is not that RIPK1 and RASAL2 form a single pathway, but that their perturbation should alter the balance between intracellular degradation and extracellular cargo disposal under defined lysosomal stress. The falsifiable predictions, testable designs, and expected interpretations are summarized in **Table 1**.

**Table 1:** Falsifiable predictions of the lysosomal-stress cargo-fate hypothesis in TNBC.

Prediction	Testable design	Expected interpretation and falsifier
RIPK1 effects extend beyond the Manzamine A model.	Compare pharmacological and genetic RIPK1 perturbation across mechanistically distinct lysosomal stressors and molecularly distinct TNBC models; where feasible, rescue with wild-type or kinase-inactive RIPK1.	Reproducible effects across stressors and models support broader involvement. Restriction to Manzamine A would define a drug- and context-specific role.
A transcriptional mechanism predicts RIPK1-dependent changes in RASAL2 expression.	Use serial RASAL2 mRNA and protein measurements, followed by promoter, chromatin or transcriptional-reporter assays.	An mRNA change preceding protein and phenotype supports transcriptional regulation. Protein-state changes without transcript change argue against it.
A post-translational mechanism predicts changes in RASAL2 protein state without corresponding transcript changes.	Measure RASAL2 phosphorylation, localization, stability and binding partners under stress and RIPK1 perturbation.	A reproducible protein-state change with stable mRNA supports post-translational regulation. No such change weakens this mechanism.
RIPK1 and RASAL2 converge functionally.	Apply single and combined perturbations and compare proximal lysosomal or autophagic readouts with downstream MVB and sEV readouts.	Non-additive effects support a shared limiting process or interaction; additive effects support parallel contributions to a common output; no interaction with distinct proximal effects supports independent arms.
Direct RASAL2-Rab27a coupling, if present, should be demonstrable independently of intact-cell co-immunoprecipitation.	Use cellular co-IP and Rab27a-GTP results as starting evidence (31). Propose purified-protein or domain-mapping binding assays and a direct Rab27a GTP-hydrolysis assay.	Purified-component binding supports direct interaction; accelerated GTP hydrolysis supports direct GAP activity. Negative results favor indirect regulation.
Lysosomal and autophagic changes precede sEV-associated release.	Sample early lysosomal pH, protease activity, membrane integrity and RIPK1-associated changes; intermediate LC3 flux, p62 turnover and autophagosome-lysosome or -MVB relationships; and later Rab27a activity, MVB docking and sEV-associated release.	A pre-death lysosomal/autophagic sequence preceding sEV increase supports cargo-fate switching. Particle increase only after marked injury argues against an active switch.
Regulated EV release is separable from death-associated particles.	At each time point assess Annexin V/PI, LDH, caspase-3/7 or PARP and, where relevant, phosphorylated MLKL (40). Normalize particle output to viable cell number and conditioning time (29).	Particle increase coupled to membrane loss, apoptosis, necrosis or necroptosis supports passive or injury-related release rather than regulated cargo-fate switching.

EV, extracellular vesicle; sEV, small extracellular vesicle; TNBC, triple-negative breast cancer.

## Experimental framework

Testing should use distinct TNBC models and mechanistically distinct lysosomal stressors within minimally cytotoxic windows. Pharmacological and genetic RIPK1 perturbation should be cross-validated, with rescue where informative; RASAL2 and RAB27A should be tested singly and in combination (30, 31, 34). Paired intracellular and extracellular readouts should share a culture interval.

Mechanistic tests should distinguish three alternatives. Transcriptional regulation predicts RASAL2 mRNA changes preceding protein and phenotype, without presuming a direct NF-kappaB link (35). Post-translational regulation predicts altered phosphorylation, localization, stability or partners without transcript change (32, 33). Functional convergence predicts shared or interacting lysosomal/autophagic and MVB/sEV effects under factorial RIPK1/RASAL2 perturbation (21, 22, 36, 37). Purified-component binding and Rab27a GTP-hydrolysis assays would test direct RASAL2-Rab27a coupling (31).

Because cargo-fate switching implies a dynamic transition, serial sampling should assess lysosomal function and RIPK1-associated changes before autophagic flux/MVB engagement and later Rab27a activity, MVB docking and sEV-associated release; timing should be adapted to each stressor.

Each time point should include membrane-integrity and apoptotic controls (29) and, where relevant, phosphorylated MLKL as a necroptotic readout (40). Regulated release requires minimal cytotoxicity, viable-cell and conditioning-time normalization, and trafficking dependence.

Patient sampling remains a secondary translational extension and cannot establish cellular temporal order (26–28, 38, 39). The proposed experimental decision framework is summarized in **Figure 2**.

**Figure 2:**
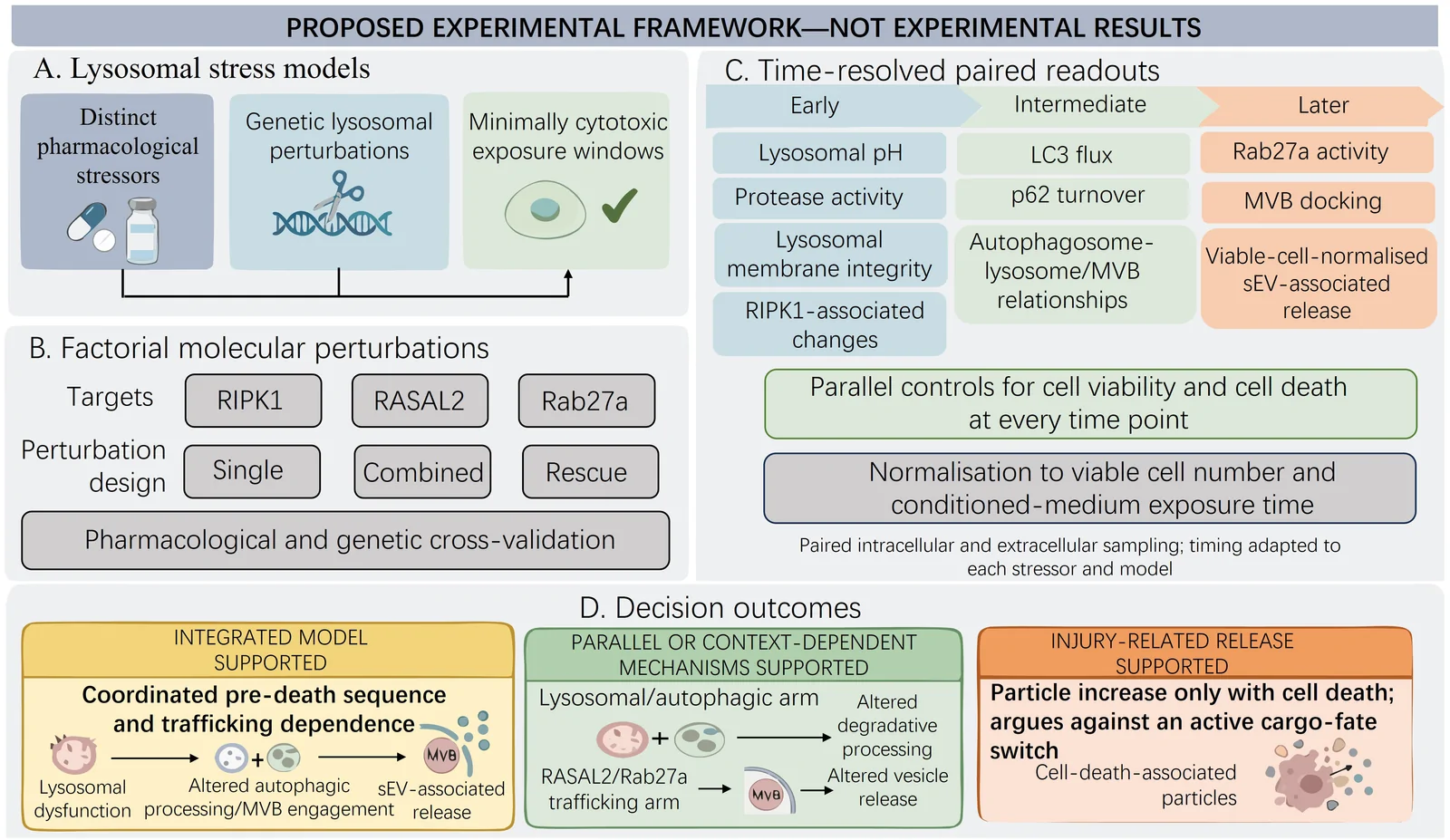
Experimental decision framework for testing the proposed cargo-fate switch. **Figure 2** presents the proposed experimental decision framework rather than completed experiments. A coordinated pre-death sequence with trafficking dependence supports the integrated model; arm-specific effects favor parallel or context-dependent mechanisms, whereas particles increasing only with cell death favor injury-related release.

## Limitations and boundary conditions

Several limitations define the scope of the proposed hypothesis. Support for RIP1 is derived primarily from Manzamine A-treated MDA-MB-231 and MCF-7 cells and has not been tested across distinct lysosomal stressors or TNBC backgrounds (30). For RASAL2-Rab27a, co-immunoprecipitation, Rab27a-GTP and combined perturbation support cellular association and functional coordination in MDA-MB-231 cells, whereas direct binding, direct GAP activity and intermediate regulators remain unresolved (31). sEV-enriched cargo is not the only extracellular route available to stressed cells (8, 11–16, 18, 34), and effects may differ among TNBC models (33, 40–43). A small proportion of dying cells can strongly influence conditioned-medium particle counts, making injury-related release an alternative explanation (29). Transcriptomic correlation is not mechanistic evidence.

If RIPK1 perturbation alters autophagic processing without changing sEV-associated release, RIPK1 may regulate only the degradative arm. If RASAL2/Rab27a perturbation alters vesicle release without affecting lysosomal function, the trafficking module may operate in parallel rather than downstream of lysosomal stress. If particle accumulation tracks cell death rather than Rab27a-dependent release, the apparent export phenotype would more likely reflect cell injury. These outcomes would not invalidate the individual observations, but they would require reformulating the model as context-dependent or parallel rather than as a unified cargo-fate switch.

## Discussion and concluding remarks

This article integrates two observations made in different models: RIP1 involvement in Manzamine A-induced secretory autophagy in breast cancer cells and RASAL2/Rab27a-associated autophagy and sEV release in MDA-MB-231 cells (30, 31). It does not establish RIPK1 as a general lysosomal-stress switch or define a linear RIPK1-RASAL2-Rab27a pathway. Testing the proposed model requires temporal ordering, experiments that distinguish transcriptional, post-translational and convergent mechanisms, and exclusion of death-associated particle release. If degradative and secretory responses separate across perturbations, the framework should be revised towards parallel or context-dependent mechanisms.

## References

[1] Sung H Ferlay J Siegel RL Laversanne M Soerjomataram I Jemal A . Global cancer statistics 2020: GLOBOCAN estimates of incidence and mortality worldwide for 36 cancers in 185 countries. CA Cancer J Clin. (2021) 71:209–49. doi: 10.3322/caac.21660 33538338

[2] Bianchini G De Angelis C Licata L Gianni L . Treatment landscape of triple-negative breast cancer: expanded options, evolving needs. Nat Rev Clin Oncol. (2022) 19:91–113. doi: 10.1038/s41571-021-00565-2 34754128

[3] Bartsch R Bagó-Horváth Z Egle D Gampenrieder SP Grünberger B Heibl S . New perspectives in the management of triple-negative breast cancer. Breast Care (Basel). (2026) 21:152–65. doi: 10.1159/000547988 41063764 PMC12503879

[4] Kimmelman AC White E . Autophagy and tumor metabolism. Cell Metab. (2017) 25:1037–43. doi: 10.1016/j.cmet.2017.04.004 28467923 PMC5604466

[5] Amaravadi RK Kimmelman AC Debnath J . Targeting autophagy in cancer: recent advances and future directions. Cancer Discov. (2019) 9:1167–81. doi: 10.1158/2159-8290.CD-19-0292 31434711 PMC7306856

[6] Kalluri R LeBleu VS . The biology, function, and biomedical applications of exosomes. Science. (2020) 367:eaau6977. doi: 10.1126/science.aau6977 32029601 PMC7717626

[7] Pegtel DM Gould SJ . Exosomes. Annu Rev Biochem. (2019) 88:487–514. doi: 10.1146/annurev-biochem-013118-111902 31220978

[8] Buratta S Tancini B Sagini K Delo F Chiaradia E Urbanelli L . Lysosomal exocytosis, exosome release and secretory autophagy: the autophagic- and endo-lysosomal systems go extracellular. Int J Mol Sci. (2020) 21:2576. doi: 10.3390/ijms21072576 32276321 PMC7178086

[9] Colletti M Ceglie D Di Giannatale A Nazio F . Autophagy and exosomes relationship in cancer: friends or foes? Front Cell Dev Biol. (2021) 8:614178. doi: 10.3389/fcell.2020.614178 33511121 PMC7835528

[10] Leidal AM Huang HH Marsh T Solvik T Zhang D Ye J . The LC3-conjugation machinery specifies the loading of RNA-binding proteins into extracellular vesicles. Nat Cell Biol. (2020) 22:187–99. doi: 10.1038/s41556-019-0450-y 31932738 PMC7007875

[11] Colombo M Moita C van Niel G Kowal J Vigneron J Benaroch P . Analysis of ESCRT functions in exosome biogenesis, composition and secretion highlights the heterogeneity of extracellular vesicles. J Cell Sci. (2013) 126:5553–65. doi: 10.1242/jcs.128868 24105262

[12] Hsu C Morohashi Y Yoshimura S Manrique-Hoyos N Jung S Lauterbach MA . Regulation of exosome secretion by Rab35 and its GTPase-activating proteins TBC1D10A-C. J Cell Biol. (2010) 189:223–32. doi: 10.1083/jcb.200911018 20404108 PMC2856897

[13] Mathieu M Martin-Jaular L Lavieu G Thery C . Specificities of secretion and uptake of exosomes and other extracellular vesicles for cell-to-cell communication. Nat Cell Biol. (2019) 21:9–17. doi: 10.1038/s41556-018-0250-9 30602770

[14] Hessvik NP Llorente A . Current knowledge on exosome biogenesis and release. Cell Mol Life Sci. (2018) 75:193–208. doi: 10.1007/s00018-017-2595-9 28733901 PMC5756260

[15] Debnath J Leidal AM . Secretory autophagy during lysosome inhibition (SALI). Autophagy. (2022) 18:2498–9. doi: 10.1080/15548627.2022.2095788 35786367 PMC9542525

[16] Solvik TA Nguyen TA Lin Y-T Marsh T Huang EJ Wiita AP . Secretory autophagy maintains proteostasis upon lysosome inhibition. J Cell Biol. (2022) 221:e202110151. doi: 10.1083/jcb.202110151 35446347 PMC9036093

[17] Li X Zhao H . Targeting secretory autophagy in solid cancers: mechanisms, immune regulation and clinical insights. Exp Hematol Oncol. (2025) 14:12. doi: 10.1186/s40164-025-00603-0 39893499 PMC11786567

[18] Guerra-Andrés M Fernández ÁChecktae Fontanil T . Exosomes, autophagy, and cancer: a complex triad. Int J Cancer. (2025) 157:405–15. doi: 10.1002/ijc.35388 40318053 PMC12141978

[19] Villarroya-Beltri C Baixauli F Mittelbrunn M Fernandez-Delgado I Torralba D Moreno-Gonzalo O . ISGylation controls exosome secretion by promoting lysosomal degradation of MVB proteins. Nat Commun. (2016) 7:13588. doi: 10.1038/ncomms13588 27882925 PMC5123068

[20] Mauthe M Orhon I Rocchi C Zhou X Luhr M Hijlkema KJ . Chloroquine inhibits autophagic flux by decreasing autophagosome-lysosome fusion. Autophagy. (2018) 14:1435–55. doi: 10.1080/15548627.2018.1474314 29940786 PMC6103682

[21] Settembre C Di Malta C Polito VA Garcia Arencibia M Vetrini F Erdin S . TFEB links autophagy to lysosomal biogenesis. Science. (2011) 332:1429–33. doi: 10.1126/science.1204592 21617040 PMC3638014

[22] Zoncu R Bar-Peled L Efeyan A Wang S Sancak Y Sabatini DM . mTORC1 senses lysosomal amino acids through an inside-out mechanism that requires the vacuolar H+-ATPase. Science. (2011) 334:678–83. doi: 10.1126/science.1207056 22053050 PMC3211112

[23] Das K Paul S Ghosh A Gupta S Mukherjee T Shankar P . Extracellular vesicles in triple-negative breast cancer: immune regulation, biomarkers, and immunotherapeutic potential. Cancers (Basel). (2023) 15:4879. doi: 10.3390/cancers15194879 37835573 PMC10571545

[24] St-Denis-Bissonnette F Khoury R Mediratta K El-Sahli S Wang L Lavoie JR . Applications of extracellular vesicles in triple-negative breast cancer. Cancers (Basel). (2022) 14:451. doi: 10.3390/cancers14020451 35053616 PMC8773485

[25] Muttiah B Ng SL Lokanathan Y Ng MH Law JX . Extracellular vesicles in breast cancer: from intercellular communication to therapeutic opportunities. Pharmaceutics. (2024) 16:654. doi: 10.3390/pharmaceutics16050654 38794316 PMC11125876

[26] Ochiya T Hashimoto K Shimomura A . Prospects for liquid biopsy using microRNA and extracellular vesicles in breast cancer. Breast Cancer. (2025) 32:10–5. doi: 10.1007/s12282-024-01563-9 38554234 PMC11717869

[27] Tamarindo GH Novais AA Frigieri BM Alves DL de Souza CA Amadeu A . Distinct proteomic profiles of plasma-derived extracellular vesicles in healthy, benign, and triple-negative breast cancer: candidate biomarkers for liquid biopsy. Sci Rep. (2025) 15:12122. doi: 10.1038/s41598-025-95232-2 40204835 PMC11982211

[28] Hyon JY Kim MW Hyun KA Yang Y Ha S Kim JY . Extracellular vesicle proteome analysis improves diagnosis of recurrence in triple-negative breast cancer. J Extracell Vesicles. (2025) 14:e70089. doi: 10.1002/jev2.70089 40545963 PMC12183389

[29] Welsh JA Goberdhan DCI O’Driscoll L Buzas EI Blenkiron C Bussolati B . Minimal information for studies of extracellular vesicles (MISEV2023): from basic to advanced approaches. J Extracell Vesicles. (2024) 13:e12404. doi: 10.1002/jev2.12404 38326288 PMC10850029

[30] Wang X Liu Y Qin H Qi G Chen X Lyu Y . RIP1 mediates Manzamine-A-induced secretory autophagy in breast cancer. Mar Drugs. (2023) 21:151. doi: 10.3390/md21030151 36976201 PMC10051755

[31] Wang X Qi G Yang K Ma L Kang Y Tang X . Rasal2 inhibits autophagic-exosomes secretion via regulating Rab27a in triple-negative breast cancer progression. J Transl Med. (2025) 23:544. doi: 10.1186/s12967-025-06530-2 40369567 PMC12079882

[32] Wang X Qian C Yang Y Liu MY Ke Y Qian ZM . Phosphorylated Rasal2 facilitates breast cancer progression. EBioMedicine. (2019) 50:144–55. doi: 10.1016/j.ebiom.2019.11.019 31759919 PMC6921363

[33] Bao Y Qian C Liu MY Jiang F Jiang X Liu H . PRKAA/AMPKα phosphorylation switches the role of RASAL2 from a suppressor to an activator of autophagy. Autophagy. (2021) 17:3607–21. doi: 10.1080/15548627.2021.1886767 33563064 PMC8632340

[34] Ostrowski M Carmo NB Krumeich S Fanget I Raposo G Savina A . Rab27a and Rab27b control different steps of the exosome secretion pathway. Nat Cell Biol. (2010) 12:19–30. doi: 10.1038/ncb2000 19966785

[35] Hayden MS Ghosh S . Shared principles in NF-kappaB signaling. Cell. (2008) 132:344–67. doi: 10.1016/j.cell.2008.01.020 18267068

[36] Perera RM Stoykova S Nicolay BN Ross KN Fitamant J Boukhali M . Transcriptional control of autophagy-lysosome function drives pancreatic cancer metabolism. Nature. (2015) 524:361–5. doi: 10.1038/nature14587 26168401 PMC5086585

[37] Rebecca VW Nicastri MC McLaughlin N Fennelly C McAfee Q Ronghe A . A unified approach to targeting the lysosome's degradative and growth signaling roles. Cancer Discov. (2017) 7:1266–83. doi: 10.1158/2159-8290.CD-17-0741 28899863 PMC5833978

[38] Cortazar P Zhang L Untch M Mehta K Costantino JP Wolmark N . Pathological complete response and long-term clinical benefit in breast cancer: the CTNeoBC pooled analysis. Lancet. (2014) 384:164–72. doi: 10.1016/S0140-6736(13)62422-8 24529560

[39] U.S. Food and Drug Administration . Pathological Complete Response in Neoadjuvant Treatment of High-Risk Early-Stage Breast Cancer: Use as an Endpoint to Support Accelerated Approval. Guidance for Industry (2020). Available online at: https://www.fda.gov/regulatory-information/search-fda-guidance-documents/pathological-complete-response-neoadjuvant-treatment-high-risk-early-stage-breast-cancer-use (Accessed March 17, 2026).

[40] Newton K . RIPK1 and RIPK3: critical regulators of inflammation and cell death. Trends Cell Biol. (2015) 25:347–53. doi: 10.1016/j.tcb.2015.01.001 25662614

[41] Yin Z Yang Q Yang S Wang Q Zhou S Li C . RIPK1 is a negative mediator in Aquaporin 1-driven triple-negative breast carcinoma progression and metastasis. NPJ Breast Cancer. (2021) 7:53. doi: 10.1038/s41523-021-00261-5 33980862 PMC8115349

[42] Olsen SN Wronski A Castano Z Dake B Malone C De Raedt T . Loss of RasGAP tumor suppressors underlies the aggressive nature of luminal B breast cancers. Cancer Discov. (2017) 7:202–17. doi: 10.1158/2159-8290.CD-16-0520 27974415 PMC6461361

[43] Feng M Bao Y Li Z Li J Gong M Lam S . RASAL2 activates RAC1 to promote triple-negative breast cancer progression. J Clin Invest. (2014) 124:5291–304. doi: 10.1172/JCI76711 25384218 PMC4348963

